# New Special Article Series: “Methodological Tutorial Series for Epidemiological Studies”

**DOI:** 10.2188/jea.JE20240387

**Published:** 2025-01-05

**Authors:** Yuri Ito

**Affiliations:** Department of Medical Statistics, Research & Development Center, Osaka Medical and Pharmaceutical University, Osaka, Japan

A New Special Article Series that introduces methodological tutorials for epidemiologists has begun in this issue. The previous Special Article Series of the *Journal of Epidemiology*, “Pitfalls and Tips for Statistical Methods in Epidemiology,” organized by Takeo Fujiwara,^1^ covered recent topics of statistical approaches for epidemiological research.^2^^–^^5^ Following the series that highlighted new approaches and advanced methods, we have started a new series focusing on practical guidance for the application of advanced methods to epidemiological studies, in cooperation with the seminars organized by the Japanese Epidemiological Association (JEA).

As the first tutorial paper, Inoue et al introduced the New Special Article Series with “Methodological Tutorial Series for Epidemiological Studies: Confounder Selection and Sensitivity Analyses to Unmeasured Confounding From Epidemiological and Statistical Perspectives,”^6^ which was based on the Pre-Seminar of the 33rd Annual Scientific Meeting of the JEA in 2023, “Selection Confounder Variables in Observational Epidemiological Studies: The Merits and Demerits of ‘Selecting and Adjusting’ Variables and Tips” organized by Tomohiro Shinozaki and Kunihiko Takahashi. In this Pre-Seminar, three young epidemiologists and statisticians introduced basic and advanced methods, the application of controlling confounders, and the rules for selecting variables. Kosuke Inoue introduced the basic idea of “confounder selection from the epidemiological perspective,” including the idea of adjusting variables based on causal diagrams, represented as directed acyclic graphs. Kentaro Sakamaki explained “confounder selection from a statistical perspective,” including pitfalls and tips of confounding selection and sensitivity analysis for unmeasured confounders. Sho Komukai showed some applied examples. The aim of the first tutorial paper of this series is to help readers to understand this important topic and to remove the barriers to application of the introduced method. In the tutorial paper, the authors included demonstration data and a script for the statistical package R (R Foundation for Statistical Computing, Vienna, Austria).

Revealing the causal association between exposure and outcome is the most important objective of epidemiological studies; however, the strategy and theory were difficult for beginners in epidemiology, and the application to real-world research data was also not so simple. Epidemiologists need to follow the recent developments in the methodology to analyze our research data adequately. Some of the JEA seminars have focused on causal inferences, including mediation and causal survival analyses. These were highly valued by the audience. This tutorial paper series will be helpful for the readers, including those who missed the seminar, to learn these methods and apply them to their own research adequately.

The JEA has long provided opportunities of education for epidemiologists, especially focusing on adequate study design and analysis to improve the quality of epidemiological studies in Japan. The JEA started the Summer Seminar in 2013 and the Pre-Seminar in 2020. Seminars on statistical and other new approaches for epidemiological research have been organized, which attracted not only members of the JEA but also a lot of researchers in various other areas, including clinical and statistical academies. Annually, about 500–800 researchers attend in-person or online seminars, especially programs focusing on statistical approaches. We recognize the need for epidemiologists and medical researchers to access continuing education on methodological and statistical topics.

As one of the Associate Editors of the *Journal of Epidemiology* and a council member of the JEA, I believe that not only young epidemiologists but also senior researchers need to understand new methods to improve the quality of epidemiological studies. I hope that this new tutorial series will reach a wide range of readers.

The long-selling textbook “*Modern Epidemiology*” has continued its contents by adding various new concepts and methods, responding to rapid global changes, including the coronavirus disease 2019 pandemic. Epidemiologists need to continue to update our knowledge of statistical and analytical methods.

The upcoming tutorial papers will introduce important topics for epidemiological studies, such as dealing with follow-up data for epidemiological studies^7^ and treatment of missing data. So far, Pre-Seminars at the Annual Scientific Meetings have been offered by request from JEA members. The JEA welcomes your ideas of organizing future seminars and tutorial papers to update the knowledge of the readers of the *Journal of Epidemiology*.
